# *Acinetobacter baumannii* Bloodstream Infections in the COVID-19 Era: A Comparative Analysis between COVID-19 and Non-COVID-19 Critically Ill Patients

**DOI:** 10.3390/microorganisms11071811

**Published:** 2023-07-14

**Authors:** Ioannis Andrianopoulos, Theodora Maniatopoulou, Nikolaos Lagos, Nikolaos Kazakos, Athanasios Papathanasiou, Georgios Papathanakos, Despoina Koulenti, Christos Kittas, Vasilios Koulouras

**Affiliations:** 1 Intensive Care Unit, University Hospital of Ioannina, 45500 Ioannina, Greece; doramaniatopoulou@yahoo.gr (T.M.); lagman75@gmail.com (N.L.); nikazakos@yahoo.com (N.K.); thanasis.papathanasiou@gmail.com (A.P.); gppthan@icloud.com (G.P.); 2 UQ Centre for Clinical Research, Faculty of Medicine, The University of Queensland, Brisbane, QLD 4029, Australia; d.koulenti@uq.edu.au; 3 Second Critical Care Department, Attikon University Hospital, Rimini Street, 12462 Athens, Greece; 4 Department of Microbiology, University Hospital of Ioannina, 45500 Ioannina, Greece; ckittas@gmail.com

**Keywords:** *Acinetobacter baumannii*, bloodstream infections, COVID-19, critically ill, ventilator-associated pneumonia, sepsis, intensive care unit, multidrug-resistant pathogens

## Abstract

The coronavirus disease (COVID-19) pandemic increased the incidence of severe infections caused by multidrug-resistant (MDR) pathogens among critically ill patients, such as *Acinetobacter baumannii* (AB), whose bloodstream infections (BSIs) have been associated with significant mortality. Whether there is any difference in outcome between COVID-19 and non-COVID-19 patients with AB BSI still remains unknown. We conducted a retrospective study comparing clinical characteristics and outcomes of COVID-19 versus non-COVID-19 critically ill patients with AB BSI. Overall, 133 patients with AB BSI (102 COVID-19, 31 non-COVID-19) were studied. The 28-day mortality rate was high and did not differ significantly (69.6% COVID-19 vs. 61.3% non-COVID-19, *p* = 0.275). Patients with septic shock had a higher mortality rate irrespective of their status with the majority of deaths occurring during the first 7 days. COVID-19 patients were more likely to have ventilator-associated pneumonia (VAP) as the source of BSI (55.8% vs. 22.3%, respectively, *p* = 0.0001) and were more likely to develop acute respiratory distress syndrome (ARDS) (78.4% vs. 48.4%, respectively, *p* = 0.001), sepsis (86.3% vs. 67.7%, respectively, *p* = 0.03), and septic shock (88.3% vs. 58.1%, respectively, *p* = 0.007) compared to the non-COVID-19 patient group. In conclusion, COVID-19 patients with *A. baumannii* BSI have a high rate of mortality and more often develop septic shock, while VAP is the main origin of their BSI.

## 1. Introduction

The emergence of the coronavirus disease (COVID-19) pandemic has led to hundreds of thousands intensive care unit (ICU) admissions, increasing ICU strain and causing excess mortality [1]. Along with the increased number in ICU admissions, an increase in the incidence of hospital-acquired infections caused by multidrug-resistant (MDR) Gram-negative pathogens has been noted [2]. Among these MDR pathogens, *Acinetobacter baumannii* (AB) appears to be one of the most common pathogen-causing such infections [3]. Carbapenem-resistant *A. baumannii* is one of the pathogens considered to be of critical importance and is included in the global priority pathogens list of MDR bacteria by the World Health Organization [WHO] and in the urgent antimicrobial resistance threats list by the Centers of Disease Control and Prevention (CDC) [4].

*A. baumannii* bloodstream infections (BSIs) had been associated with high mortality rates among critically ill patients in a significant number of reports before the onset of the COVID-19 pandemic [5]. Initial reports, early in the pandemic, showed an increased prevalence of MDR *A. baumannii* co-infection in COVID-19 critically ill patients that was associated with high mortality rates [6,7]. This high mortality rate was also clinically noted during the first three years of the pandemic in our ICUs, along with an increased prevalence of *A. baumannii* BSIs. To date, little evidence exists on whether COVID-19 infection increases the mortality rate from that of AB BSIs, and also, on whether COVID-19 patients with AB BSIs, have any differences in their clinical characteristics and in the outcomes compared to non-COVID-19 patients. 

The purpose of our study was first, to identify the impact of AB BSI on mortality rates in our COVID-19 ICU population, second, to assess whether there is any difference in terms of AB BSI’s outcomes between COVID-19 and non-COVID-19 critically ill patients, and third, to assess for any differences in clinical characteristics and complications between the COVID-19 and non-COVID-19 populations.

## 2. Materials and Methods

### 2.1. Study Design

We performed a retrospective, observational cohort study including all adult patients admitted in the two intensive care units of Ioannina University Hospital, a 900-bed tertiary hospital in Ioannina, Greece, that had a positive blood culture for *A*. *baumannii* (the recruitment period was from March 2020 to March 2023). The institutional review board of the University Hospital of Ioannina (protocol number 348/2023) approved the anonymized data collection and waived the need for informed consent due to the observational, retrospective nature of the study.

### 2.2. Data Collection and Patient Groups

We recorded information on: age; sex; comorbid conditions, including the Charlson’s comorbidity index score (CCI); and severity, including the Sequential Organ Failure Assessment (SOFA) and the Acute Physiologic Assessment and Chronic Health Evaluation (APACHE) II scores on admission, as well as the SOFA score 24 h before sepsis onset, on the day of sepsis onset and on days 3 and 7 after sepsis onset. We also recorded complications, including sepsis, septic shock, acute kidney injury (AKI), acute respiratory distress syndrome (ARDS), coagulopathy, hepatic dysfunction, need for continuous renal replacement therapy (CRRT), 7-day outcomes, including microbiological cure, resolution of sepsis and vasopressor discontinuation, all-cause 28-day mortality and all-cause hospital mortality, ICU length of stay (LOS), duration of mechanical ventilation, and association of death with the A. bloodstream infection. For patients with more than one episode of AB BSI, only the first one was taken into consideration and data were recorded according to this first episode.

We categorized patients into two groups. Patients with a current COVID-19 infection and a positive polymerase chain reaction (PCR) test for severe acute respiratory syndrome coronavirus 2 (SARS-CoV-2) formed group A and patients without COVID-19 infection and a negative PCR SARS-CoV-2 test formed group B. The vaccination status of each COVID-19 patient was recorded.

### 2.3. Study Outcomes

The primary outcome of the study was all-cause 28-day mortality. The secondary outcomes included the length of stay in the ICU and in the hospital, the duration of mechanical ventilation, the time from septic shock to death for those that died due to the *A. baumannii* infection, the 7-day all-cause mortality, the 7-day microbiological cure, the 7-day resolution of sepsis, the 7-day need for vasopressors, the need for CRRT, the development of sepsis, and the development of organ failures.

### 2.4. Definitions

Sepsis and septic shock were defined based on the third international consensus definitions for sepsis and septic shock [8]. ARDS was defined based on the Berlin definition; for patients already with ARDS, a worsening of the severity stage of ARDS and worsening of the bilateral lung infiltrates were considered as new ARDS complications of the AB BSI [9]. Acute kidney injury (AKI) was defined and staged based on the guidelines of ‘Kidney Disease: Improving Global Outcomes’ (KDIGO) [10]. Coagulopathy was defined as, either platelets < 150,000/μL, or INR > 1.4. Hepatic dysfunction was defined as a value of total bilirubin greater than 2 mg/dL [11]. Microbiological cure was defined as a negative for AB follow-up blood culture by day 7. Death was attributed to *A. baumannii* if at least two attending physicians decided that the patient died of septic shock, the cause of sepsis was the *A. baumannii* infection causing the studied BSI, symptoms were not resolved and white blood cell (WBC) count and C-reactive Protein (CRP) did not return to their normal limits. Resolution of sepsis was defined as a SOFA score on day 7 from sepsis SOFA less or equal to the SOFA score calculated 24 h before sepsis plus one more point. As data were collected retrospectively, ventilator-associated pneumonia (VAP) was defined using the Centers for Disease Control and Prevention (CDC) criteria [12].

### 2.5. Statistical Analysis

Normal distribution of continuous variables was evaluated using the Kolmogorov–Smirnov test and presented as mean ± standard deviation, or median, min–max values as appropriate. Comparisons between the two study groups were performed using the Mann–Whitney test or the *t*-test, as appropriate for the continuous variables whereas, chi-square tests using *p*-values as extracted from Fisher’s exact test were used for categorical variables. The assumption concerning the proportionality of hazards was graphically assessed and met for all covariates and Kaplan–Meier survival plots of matched groups were constructed and compared by using the log-rank test. Significance was defined as a *p*-value < 0.05 in all cases. SPSS^®^ 21.0 (Chicago, IL, USA) was used for all analyses.

## 3. Results

Overall, 133 critically ill patients out of 569 (286 COVID-19 patients, 263 non-COVID-19 patients) developed at least one episode of bloodstream infection from *A. baumannii* and were included in our study. Out of these 133 patients (93 men, 69.9% and 40 women, 30.1%), 102 were COVID-19 (Group A) and 31 were non-COVID-19 (group B). Mean age of the study sample was 67.1 ± 12.4 years.

### 3.1. Baseline Characteristics of COVID-19 and Non-COVID-19 Patients with A. baumannii Bloodstream Infection

Baseline characteristics of COVID-19 and non-COVID-19 patients that developed an episode of AB BSI are shown in Table 1. Non-COVID-19 patients (Group B) had a worse APACHE II (23.8 ± 9.5 vs. 20.72 ± 5.4, *p* = 0.01) and a worse median SOFA score (8 vs. 5, respectively, *p* = 0.001). There were no differences in age, sex, mechanical ventilation, or comorbidities in the two study groups (Table 1).

Enrollment of patients in the study per study year and patient group is shown in Supplementary Table S1.

### 3.2. Clinical Characteristics of A. baumannii Bloodstream Infections BSI in COVID-19 and Non-COVID-19 Patients

The clinical characteristics of the AB BSIs in the COVID-19 and non-COVID-19 patients are shown in Table 2. Patients with COVID-19 had more often than non-COVID-19 patients pneumonia as the source of bloodstream infection (55.8% vs. 22.5%, respectively, *p* = 0.0001), they received more often corticosteroids (98% vs. 48.4%, respectively, *p* = 0.0001) and tocilizumab (63.7% vs. 0%, respectively, *p* = 0.0001), developed more often sepsis (86.3% vs. 67.7%, respectively, *p* = 0.03), septic shock (88.3% vs. 58.1%, respectively, *p* = 0.007), and ARDS (78.4% vs. 48.4%, respectively, *p* = 0.001) (Table 2).

### 3.3. Outcome of COVID-19 and Non-COVID-19 Patients with Bloodstream Infection from A. baumannii

The outcomes of COVID-19 and non-COVID-19 critically ill patients that developed a BSI from *A. baumannii* are shown in Table 3. All-cause 28-day mortality post-BSI as well as an all-cause hospital mortality rate was high among both the COVID-19 and the non-COVID-19 patient groups (69.6% vs. 61.3%, and 74.5% vs. 74.2%, respectively), but there was no statistically significant difference between them. The majority of deaths in the COVID-19 group (70/133 patients, 52.6%) were attributed to *A. baumannii* infection. On the one hand, ICU-LOS, duration of mechanical ventilation and time from shock to death (4 days) were similar in the two groups. On the other hand, 7-day outcomes varied significantly as non-COVID-19 patients were more likely to be off-vasopressors (32.3% vs. 9.8%, respectively, *p* = 0.007) and were more likely to have resolution of sepsis (32.3% vs. 12.7%, respectively, *p* = 0.027) by day 7 after the onset of AB BSI (Table 3). 

**Figure 1:**
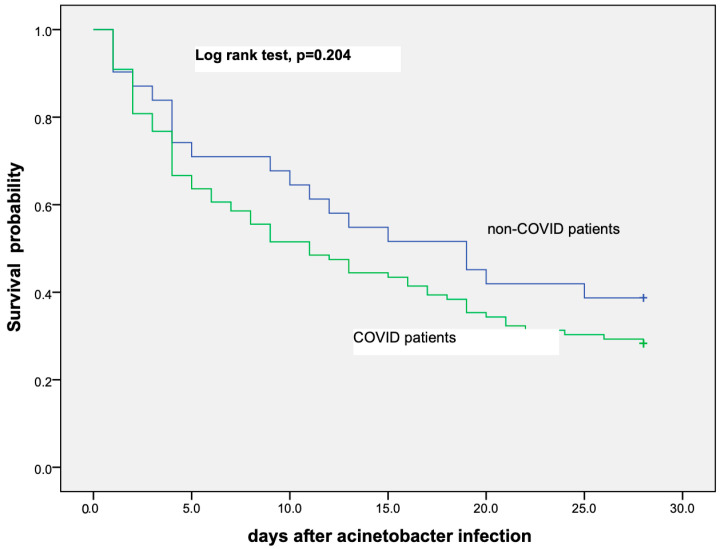
Kaplan–Meier curve showing probability of survival among COVID-19 and non-COVID-19 critically ill patients that have developed *A. baumannii* bloodstream infection.

The probability of survival for the first 28 days after a positive *A. baumannii* BSI was similar in both COVID-19 and non-COVID-19 critically ill patients as is shown in the Kaplan–Meier curve in Figure 1.

Only 16 out of the 102 (15.6%) COVID-19 critically ill patients were vaccinated against SARS-CoV-2. Mortality was high in this subgroup of patients (81.25%).

### 3.4. Outcome of COVID-19 and Non-COVID-19 Patients with Septic Shock from A. baumannii Bloodstream Infection

Both 28-day all-cause mortality and 7-day all-cause mortality in COVID-19 and non-COVID-19 critically ill patients with *A. baumannii* bloodstream infection are shown in Table 4. Patients with septic shock had a statistically significant higher rate of mortality both at day 7 as well as at day 28 post-positive blood culture compared to patients without septic shock. There was no statistically significant difference between both 7-day as well as 28-day mortality rates among the COVID-19 and the non-COVID-19 patients. Finally, more than 50% of deaths of patients occurred early in the first 7 days (in 59.15% of COVID-19 and in 52.63% of non-COVID-19 patients, respectively) (Table 4).

**Figure 2:**
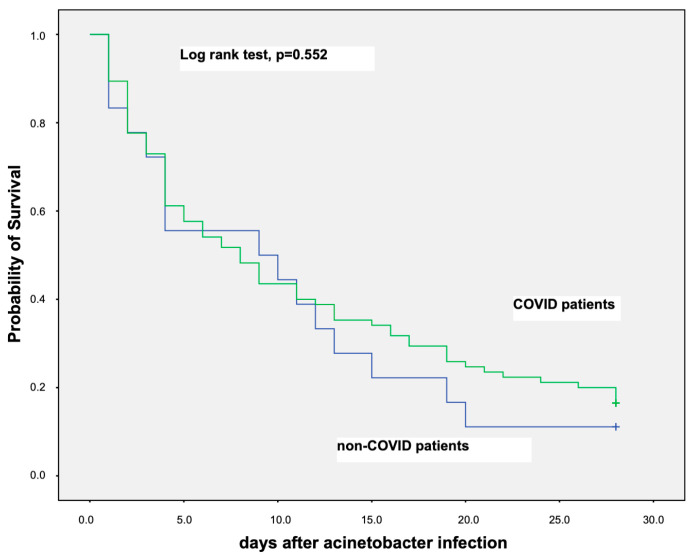
Kaplan–Meier curve showing probability of survival for the first 28 days among COVID-19 and non-COVID-19 critically ill patients that have developed septic shock due to *A. baumannii* bloodstream infection.

Probability of survival for the first 28 days among COVID-19 and non-COVID-19 critically ill patients with septic shock from *A. baumannii* BSI was similar as is shown in the Kaplan–Meier curve in Figure 2.

## 4. Discussion

This study of 133 (102 COVID-19 and 31 non-COVID-19, respectively) critically ill patients with *A. baumannii* BSI showed a high mortality rate (overall 28-day mortality was 67.6% for both groups). No statistically significant difference in 28-day all-cause mortality was noted between the COVID-19 and non-COVID-19 patients (69.6% vs. 61.3%, respectively). More than 50% of patients died because of the *A. baumannii* infection (52.6%) and more than 50% of deaths occurred in the first 7 days post-bacteremia. Septic shock was associated with high mortality in both the COVID-19 and non-COVID-19 patient groups. Patients with COVID-19 and AB BSIs had worse severity scores on admission (APACHE II score, SOFA score), had more often VAP as the source of BSI, and developed more often sepsis and septic shock compared to the non-COVID-19 patients.

Our study showed a high mortality rate in both study groups similar to other studies [7,13,14,15,16]. Possible explanations for this high mortality include the high percentage of septic shock (88.6% in the COVID-19 and 58.1% in the non-COVID-19 group, respectively), the high incidence of organ failure (e.g., ARDS 78.4% in COVID-19 and 48.4% in non-COVID-19, respectively), AKI stage 3 (25.5% in COVID-19 and 32.2% in non-COVID-19, respectively), the high percentage of VAP (55.5% in the COVID-19 population) and the high severity of illness (the median SOFA score on day 1 of sepsis was 9) that are known risk factors associated with increased mortality [17]. In addition, carbapenem-resistant *A. baumannii* (CRAB) is a pathogen with difficult-to-treat resistance (DTR) and sometimes pandrug resistance (PDR), and severe infections of these pathogens are associated with high mortality [17], while treatment is reliant on combinations of antibiotics [18,19,20]. Possibly, new combinations [21] or new antibiotics, such as cefiderocol may improve the outcomes [13,22].

Our study did not show a statistically significant difference in mortality between the COVID-19 and the non-COVID-19 group. These results are consistent with the other two trials that performed a direct comparison of COVID-19 and non-COVID-19 critically ill patients by Russo et al. and Alenazzi et al. [14,15]. On the one hand, despite differences in the study group populations, mortality was high in both groups across all three studies. On the other hand, in our study, mortality was high in septic shock patients in both groups, while non-septic shock patients had a significantly lower rate of mortality. These two findings, the high mortality rate of septic shock patients and the lack of statistically important differences between the COVID-19 and the non-COVID-19 patient groups, might possibly signify that in those cases where the patients develop septic shock, the main driving force of mortality is the septic shock from the BSI rather than the underlying ICU admission cause of the patient, and that whatever the increased COVID-19 mortality risk is, it gets cancelled out by the mortality risk of septic shock. This result is consistent with our previous study on *A. baumannii* BSI where patients with septic shock had a very high mortality rate [5].

Another important finding in our study was the high 7-day mortality post-positive blood culture for *A. baumannii*. More than 50% of deaths occurred within the first 7 days from a positive blood culture for AB. This early mortality can be more directly attributed to *A. baumannii* BSI and possibly better reflects the burden of mortality of the BSI. Unfortunately, most studies use 28-day mortality or hospital mortality to assess the outcome of BSI. Some studies have used 14-day mortality as the outcome and fewer studies have used the 7-day outcome [23,24]. Our results on 7-day mortality are consistent with the results of the later studies.

In addition, our study showed that COVID-19 critically ill patients with AB BSI have different clinical characteristics compared to non-COVID-19 patients. In particular, the source of BSI is mainly respiratory rather than primary and patients more often develop sepsis, septic shock, and ARDS. Bacterial superinfections of the lung are more common in COVID-19 patients than non-COVID-19 patients, but their incidence varies significantly depending on the study population and may vary in critically ill patients from 32–95% [16,25,26]. The cause of this increased incidence of superinfections is likely multifactorial, and is associated with different factors such as immunoparalysis, microbiome dysbiosis, evasion of host-defenses as well as, among others, prolonged mechanical ventilation, sedation, and ICU length of stay [26]. Moreover, the attributable mortality of VAP in COVID-19 patients is increased [27]. Finally, the high incidence of sepsis and septic shock observed in critically ill patients with AB BSI is worth mentioning, as most likely, it is one of the driving forces for mortality in DTR infections [19].

Further studies are required to focus on the identification of risk factors for mortality from AB BSIs, as well as on interventions on prevention and earlier diagnosis of VAP and sepsis. Finally, further research is required for new therapeutic strategies and novel antibiotics for DTR AB.

### Limitations-Strengths

Our study has some limitations. First, it is a retrospective, single-center study and therefore subject to the inherent limitations of such types of studies. In addition, there were some missing data on patients’ comorbidities and complications, but these were evenly distributed both in COVID-19 and non-COVID-19 patients. Another limitation is the uneven size of the two compared groups as the non-COVID-19 group is smaller, nevertheless this reflects the increased prevalence of AB BSIs in COVID-19 critically ill patients.

However, our study has several strengths. To the best of our knowledge, our study is the largest on AB BSI in COVID-19 critically ill patients and the largest that compares AB BSI in COVID-19 and non-COVID-19 patients. Also, our study is one of the few that examines 7-day post-bloodstream infection and the first to one that addresses it in *A. baumannii* bloodstream in COVID-19 critically ill patients. The study of early mortality in patients with BSI offers more information on the mortality directly related to the BSI infection per se.

## 5. Conclusions

*Acinetobacter baumannii* bloodstream infections are associated with high mortality rates in both COVID-19 and non-COVID-19 critically ill patients. Patients with *A. baumannii* BSI, irrespective of their COVID-19 status, experience high mortality if they develop septic shock. COVID-19 critically ill patients with AB BSI more commonly have pneumonia as the source of BSI and more often develop sepsis and septic shock.

## Figures and Tables

**Table 1:** Baseline characteristics of the COVID-19 and non-COVID-19 critically ill patients with an *A. baumannii* bloodstream infection.

Parameter	Group A COVID-19 Patients (*n* = 102)	Group B Non-COVID-19 Patients (*n* = 31)	*p*-Value
Age (mean ± SD)	66.2 ± 14.8	67.3 ± 11.6	NS *
Gender (female n, %)	32 (31.4%)	8 (25.8%)	0.658
CCI * [median (min–max)]	3 (0–11)	4 (0–10)	0.326
APACHE II (mean ± SD)	20.72 ± 5.4	23.8 ± 9.5	0.01
SOFA * on admission [median (min–max)]	5 (1–13)	8 (3–17)	0.001
Patients on mechanical ventilation at day of ICU admission, No. (%)	90 (88.2%)	24 (77.4%)	0.185
Co-morbidities			
Diabetes mellitus, No. (%)	29 (28.4%)	8 (25.8%)	0.824
Immunosuppressive treatment, No. (%)	7 (6.9%)	1 (3.2%)	0.680
Chronic corticosteroid therapy, No. (%)	1 (1%)	4 (12.1%)	0.011
Heart failure, No. (%)	7 (6.9%)	2 (6.5%)	NS
Ischemic Heart Disease, No. (%)	13 (12.7%)	6 (19.4%)	0.384
Chronic Kidney Disease, No. (%)	7 (6.9%)	2 (6.5%)	NS
Liver Cirrhosis, No. (%)	2 (2%)	0	NS
COPD *, No. (%)	9 (8.8%)	4 (12.1%)	0.5
Malignancy, No. (%)	8 (7.8%)	4 (12.1%)	0.473

* CCI, Charlson Comorbidity Index; SOFA, Sequential Organ Failure Assessment score; COPD, Chronic Obstructive Pulmonary Disease; NS, not significant.

**Table 2:** Clinical characteristics and complications of *A. baumannii* bloodstream infections among the COVID-19 and the non-COVID-19 study groups.

Parameter	Group A COVID-19 Patients (*n* = 102)	Group B Non-COVID-19 Patients (*n* = 31)	*p*-Value
Site of infection			
Primary, No. (%)	27 (26.5%)	6 (19.4%)	0.0001 ^∫^
Pneumonia, No. (%)	57 (55.8%)	7 (22.5%)	
Urinary tract, No. (%)	0	0	
Unspecified, No. (%)	15 (14.8%))	13 (41.9%)	
Other, No. (%)	3 (2.9%)	5 (16.1%)	
SOFA * score on day 1 of sepsis, median (min–max)	9 (2–16)	9 (2–17)	NS *
Patients receiving appropriate empiric therapy on day 1 of sepsis, No. (%)	73 (71.6%)	17 (54.8%)	0.124
Pharmacological therapy other than antibiotics			
Tocilizumab, No. (%)	65 (63.7%)	0	0.0001
Steroids, No. (%)	100 (98%)	15 (48.4%)	0.0001
Dose of corticosteroids > 12 mg dexamethasone per day or equivalent, No. (%)	4 (3.9%)	7 (22.6%)	0.003
Receiving corticosteroids for more than 14 days, No. (%)	49 (48%)	9 (29%)	0.067
Characteristics of *A. baumannii* strain, No. (%)			
PDR *	24 (23.5%)	3 (9.7%)	0.126
Colistin Sensitive	36 (35.3%)	10 (32.3%)	0.832
Tigecycline Sensitive	67 (65.7%)	26 (83.9%)	0.073
Only sensitive to Tigecycline	30 (29.4%)	14 (45.2%)	0.128
Complication(s) related to *A. baumannii* infection, No. (%)			
Sepsis	88 (86.3%)	21 (67.7%)	0.03
Shock	85 (88.3%)	18 (58.1%)	0.007
ARDS *	80 (78.4%)	15 (48.4%)	0.001
AKI *	42 (41.2%)	17 (54.8%)	0.203
KDIGO * worst stage, No. (% among all patients in the group, % among patients with AKI * in the group)			
Stage 1	9 (8.8%, 21.4%)	2 (6.5%, 11.8%)	0.047
Stage 2	7 (6.9%, 16.7%)	5 (16.1%, 29.4%)	
Stage 3	26 (25.5%, 83.8%)	10 (32.2%, 58.8%)	
Patients receiving CRRT *, No. (%)	11 (10.8%)	4 (12.9%)	0.746
Cardiomyopathy ^∞^, No. (%)	9 (8.8%)	2 (6.5%)	0.727
Coagulopathy ^∞^, No. (%)	62 (60.8%)	16 (51.6%)	0.294
Liver Dysfunction ^∞^, No. (%)	45 (44.2%)	11 (35.5%)	0.406

* Abbreviations: SOFA, Sequential Organ Failure Assessment Score; PDR, Pandrug-resistant; ARDS, Acute Respiratory Distress Syndrome; AKI, Acute Kidney Injury; KDIGO, ‘Kidney Disease: Improving Global Outcomes’; CRRT, Continuous Renal Replacement Therapy; NS, not significant. ^∫^ *p*-value refers to difference between different sites of infection both in COVID-19 and non-COVID-19 patients. ^∞^ Coagulopathy was defined as International Normalized Ratio (INR) > 1.4 or Platelet count < 150,000/μL. Hepatic dysfunction was defined as a value of Total Bilirubin > 2 mg/dL. Cardiomyopathy was defined as new onset left ventricular systolic dysfunction at the time of sepsis.

**Table 3:** Outcomes of *A. baumannii* bloodstream infections in the COVID-19 and the non-COVID-19 critically ill patients.

Parameter	Group A COVID-19 Patients (*n* = 102)	Group B Non-COVID-19 Patients (*n* = 31)	*p*-Value
Mortality outcomes			
All-cause hospital mortality ^∫^, (%)	76 (74.5%)	23 (74.2%)	NS *
Deaths related to *A. baumannii* BSI *^€^, No. (%)	56 (54.9%)	14 (45.2%)	0.787
28-day all-cause hospital mortality ^∫^, No. (%)	71 (69.6%)	19 (61.3%)	0.275
7-day all-cause hospital mortality ^∫^, No. (%)	42 (41.6%)	10 (32.3%)	0.405
Secondary outcomes			
Free of vasopressors by day 7 ^∞^	10 (9.8%)	10 (32.3%)	0.007
Microbiological cure by day 7 ^∞^	24 (23.5%)	11 (35.5%)	0.244
Resolution of sepsis by day 7 ^∞^	13 (12.7%)	10 (32.3%)	0.027
Days from septic shock onset to death, median (min–max)	4 (0–122)	4 (0–19)	NS
Days in ICU, median (min–max)	20 (2–154)	24 (1–233)	NS
Days in hospital, median (min–max)	31 (7–263)	42 (0–279)	NS
Days on mechanical ventilation, median (min–max)	20 (1–115)	20 (0–115)	NS

* NS, not significant; BSI, blood stream infection. ^∫^ 28-day and 7-day all-cause hospital mortality rates were defined as death from the day of a positive blood culture for *A. baumannii* up until 28 or 7 days post the positive blood culture result. ^€^ Death was attributed to A. *baumannii* if at least two attending physicians decided that the patient died of septic shock. ^∞^ Free of vasopressors by day 7 was defined as the patient not receiving any vasopressors on the 7th day post the positive blood culture for *A. baumannii.* Microbiological cure was defined as a negative for *A. baumannii* follow-up blood culture by day 7. Resolution of sepsis was defined as a SOFA score on day 7 from sepsis SOFA less or equal to the SOFA score calculated 24 h before sepsis plus one more point.

**Table 4:** Mortality rates between the two study groups at 7 days and 28 days from the onset of *A. baumannii* blood stream infection relative to the development of septic shock.

Parameter	Group A COVID-19 Patients (*n* = 102)	Group B Non-COVID-19 Patients (*n* = 31)	*p*-Value
28-day all-cause hospital mortality ^∞^, No. (%)	71 (69.6%)	19 (61.3%)	0.275
28-day mortality for patients with or without septic shock			0.0001 *
Patients with septic shock, No. (%) ^€^	69 out of 85 (81.2%)	16 out of 18 (88.9%)	
Patients without septic shock, No. (%) ^€^	2 out of 17 (11.8%)	3 out of 13 (23.1%)	
7-day all-cause hospital mortality ^∞^, No. (%)	42 (41.6%)	10 (32.3%)	0.405
7-day mortality for patients with or without septic shock			0.0001 *
Patients with septic shock, No. (%) ^€^	42 out of 85 (49.5%)	9 out of 18 (50%)	
Patients without septic shock, No. (%) ^€^	0 out of 17 (0%)	1 out of 13 (7.7%)	

* *p*-Value refers to difference between the septic and non-septic shock patients both in COVID-19 and non-COVID-19 patients. ^∞^ 28-day and 7-day all-cause hospital mortality rates were defined as death from the day of a positive blood culture for *A. baumannii* up until 28 or 7 days post-positive blood culture result. ^€^ Percentages refer to the number of deaths in each subgroup (with or without septic shock, respectively) population.

## Data Availability

The datasets used and/or analyzed during the current study are available from the corresponding author on reasonable request.

## Supplementary Materials

The following supporting information can be downloaded at: https://www.mdpi.com/article/10.3390/microorganisms11071811/s1, Table S1: Enrollment of patients in the study per study year and group.
