# Supplementary material for: Acinetobacter baumannii Bloodstream Infections in the COVID-19 Era: A Comparative Analysis between COVID-19 and Non-COVID-19 Critically Ill Patients
Source: Microorganisms. 2023 Jul 14;11(7):1811. doi: 10.3390/microorganisms11071811 (PMC10383443; doi:10.3390/microorganisms11071811)
Supplement: Supplementary file 1 [file microorganisms-11-01811-s001.zip › microorganisms-2471767-supplementary.pdf]

Supplementary Table S1. Enrollment of patients in the study per study year and group.

	2020	2021	2022	2023	Total
COVID-19 patients	**15**	**47**	**40**	**0**	**102**
Non-COVID-19 patients	**8**	**7**	**11**	**5**	**31**
